# An Exploratory Study Testing Autonomic Reactivity to Pain in Women with Sensory Over-Responsiveness

**DOI:** 10.3390/brainsci10110819

**Published:** 2020-11-05

**Authors:** Tami Bar-Shalita, Nurit Ben-Ziv, Yelena Granovsky, Irit Weissman-Fogel

**Affiliations:** 1 Department of Occupational Therapy, School of Health Professions, Faculty of Medicine, Tel Aviv University, Tel Aviv 6997801, Israel; tbshalita@post.tau.ac.il (T.B.-S.); benzivng@gmail.com (N.B.-Z.); 2 Sagol School of Neuroscience, Tel Aviv University, Tel Aviv 6997801, Israel; 3 Department of Neurology, Rambam Health Care Campus and the Laboratory of Clinical Neurophysiology, Faculty of Medicine, Technion, Haifa 3525433, Israel; y_granovsky@rambam.health.gov.il; 4 Physical Therapy Department, Faculty of Social Welfare and Health Sciences, University of Haifa, Haifa 3498838, Israel

**Keywords:** sensory over-responsiveness, sensory integration dysfunction, vagal tone, pain, healthy, pain psychophysics, autonomic nervous system

## Abstract

Background: Difficulty modulating sensory input related to multi-sensory integration dysfunction, specifically the sensory over-responsive (SOR) type, is associated with psychological distress and hyperalgesia in children and adults. Scares reports suggest atypical autonomic nervous system (ANS) reactivity to innocuous sensory stimuli in children with SOR. Thus, the ANS may contribute to sensory stimuli responses and psychological distress. This exploratory study aimed to characterize the ANS reactivity to single and dual pain stimulation, and in relation to psychological distress in adults with SOR. Methods: Healthy women with SOR (*n* = 9) vs. without SOR (*n* = 9) underwent two runs of single pain stimulation and a third run comprised of dual pain stimulation. Pain was self-rated, while heart rate variability was measured and analyzed in the time and frequency domains. In addition, questionnaires assessing anxiety and somatization were utilized. Results: While controls demonstrated a vagal tone withdrawal (root mean square of successive differences in R-R-intervals; (RMSSD)) *p* = 0.029 from base-line to the third run, this was absent in the SOR group. However, no group differences were found in pain ratings. Furthermore, groups differed in the correlations between R-R mean and the level of both anxiety (*p* = 0.006) and somatization (*p* < 0.001); while in the SOR group, higher levels of anxiety and somatization correlated with shorter R-R intervals, the opposite was found in the control group. Conclusions: This is the first study to demonstrate in women with SOR atypical vagal tone reactivity to challenging pain load. Vagal tone reactivity is related to both pain ratings and psychological distress.

## 1. Introduction

Difficulty modulating sensory input, specifically the sensory over-responsiveness (SOR) type, is characterized by increased cortical neural responses to non-painful sensory stimuli [1,2,3,4,5,6]. Individuals with SOR experience environmental sensory input as abnormally irritating or painful, which severely limits daily functioning and quality of life [5,7]. Environmental sensory challenges evoke compromised homeostatic processes which require autonomic nervous system (ANS) adjustments through vagal withdrawal and sympathetic activity [8,9]. Children with SOR show an increase in sympathetic nervous system reactivity in response to sensory stimuli [10], a low vagal tone at baseline and deficient vagal withdrawal, which was associated with their symptom severity [11,12]. ANS activity is suggested as a biomarker for sensory modulating difficulties [11], which to date has not been tested in adults.

Over time, adults with SOR develop coping strategies and adaptive behaviors in order to function efficiently within their daily environments [13]. However, under sensory loaded or sensory threatened environments, these strategies are no longer efficient [14]. Therefore, in order to explore behavioral responses and ANS activity and reactivity, it is necessary to induce high demand stimuli, namely pain. Indeed, individuals with SOR behaviorally demonstrate hypersensitivity responses (hyperalgesia) to experimental and everyday pain stimuli [15,16,17,18].

Pain as a threatening stimulus always activates the ANS, which in turn modulates the pain responses. Specifically, the parasympathetic nervous system, i.e., the vagus nerve, activates the pain descending pathways and thus induces analgesic effects [19,20]. In synchrony, the sympathetic nervous system is also activated; in healthy individuals, due to pain stimulus, the sympathetic nervous system is activated and suppresses pain, and yet in pathological conditions, it augments pain [21]. Finally, psychological distress, which characterizes individuals with SOR [22], may further amplify pain sensitivity as well as increasing sympathetic nervous system reactivity, and has lasting effects on baseline ANS activity [23].

Taken together, the ANS activity and reactivity may add to current knowledge explaining behavioral responses in individuals with SOR. Thus, in this exploratory study, we aimed to investigate the ANS activity at baseline and in response to single and more challenging dual pain stimulation in adults with SOR, and in relation to psychological distress.

## 2. Materials and Methods

The experimental protocol was approved by the Ethics Committee of Rambam Health Care Campus (#3075), and all participants provided informed consent. This is part of a cross sectional comparative research project that has been partly reported [18] and designed in accordance with the research recommendations for experiments measuring ANS activity [24].

### 2.1. Participants

Adhering to the reported sex variance in ANS measures [25], healthy females ≥18 years with SOR (*n* = 9; study group) and without sensory modulating difficulties (*n* = 9; control group) participated in this study. Participants with SOR had been referred by an occupational therapist, with expertise in sensory modulation dysfunction following a comprehensive interview. The participants in the control group were recruited from the hospital staff and university students.

Exclusion criteria for both groups included: psychological, metabolic, and neurological diseases; pregnancy; and acute or chronic pain conditions, based on an interview and a medical questionnaire. Inclusion criteria for the study group: scoring above the normal cut-off scores (mean ± 2 SD; >2.39) on the Sensory Responsiveness Questionnaire—Intensity scale (SRQ-IS), Aversive sub-scale [26] (see Section 2.4.1). Inclusion criteria for the control group: scoring below mean ± 1 SD in both SRQ-IS (<2.13 in the Aversive and <2.43 in the Hedonic scales). The average SRQ-Aversive scores were 2.76 (SD: 0.48) for the SOR group and 1.61 (0.15) for the control group (*p* > 0.001), the latter confirming group allocation.

### 2.2. Pain Assessment

Two levels of challenging pain stimuli were applied: (i) a single individually tailored pain stimulation comprised of 14 single heat pain stimuli, and (ii) dual pain stimulation comprised of the latter in addition to another pain stimulation (i.e., hot water bath) as described below.

Prior to the stimuli applications, participants were informed that they will receive heat stimuli at intensities that do not cause harm or damage and are safe based on the FDA requirements. The heat stimuli were delivered using the Contact Heat-Evoked Potential Stimulator, a computerized thermal stimulator (Medoc Ltd. Advanced Medical Systems, Ramat Yishai, Israel). This device generates heat stimuli using a flat disk probe contact surface with a round cutaneous area of 572.5 mm^2^ (27 mm in diameter). The temperature used was individually tailored to induce moderate pain magnitude of 50/100 (pain-50) on a numerical pain scale (NPS: 0 = not painful at all; 100 = most imaginable pain) [27]. In detail, a series of paired stimuli was applied starting from a baseline temperature of 45 °C and reaching a max. of 51 °C, gradually increasing/decreasing by 1 °C, with an onset-to-onset inter-stimulus-interval of 8 s. For each stimulus, subjects were required to rate the pain intensity. When paired stimuli were rated 50, the individual temperature was attained and was utilized through this experimental study [18].

Three runs, each consisting of 14 contact heat stimuli delivered by Contact Heat-Evoked Potential Stimulator, were applied to the volar aspect of the forearm of the dominant hand at the individual tailored pain-50 (single stimulation). In the 3rd run, the pain stimuli were given simultaneously with the immersion of the non-dominant hand in a painful hot (46.5 °C) water bath (Heto Cooling Bath, Jouan Nordic A/S, Allerod, Denmark) (dual stimulation). Subjects were asked to rate their pain intensity induced by the Contact Heat-Evoked Potential Stimulator after each stimulus using the NPS.

### 2.3. Heart Rate Variability (HRV) Assessment

The experiment included the following three phases: baseline activity, reactivity to pain, and recovery. Recording and analysis of the HRV were conducted via the SUEmpathy100 (S^®^; SUESS Medizin-Technik, Aue, Germany). The real-time electrocardiogram (ECG) signal was transferred via Bluetooth to a computer program (SUEmpathy100, version SUE1-4.36j; SUESS Medizin-Technik, Aue, Germany). Off-line analysis included time and frequency domain analyses of the HRV. The first step included a visual inspection of the data and removal of epochs with signal artifacts. For the time domain, the following variables were included: R-R interval mean (ms), percentage of successive normal sinus R-R intervals more than 50 ms (PNN50), and the root mean square of successive differences (RMSSD); the latter two are measures of vagal tone. For the frequency domain we measured low-frequency power (LF: 0.04–0.15 Hz, reflecting sympathetic and parasympathetic activity), high-frequency power (HF; 0.15–0.40 Hz, reflecting parasympathetic activity), and LF/HF (reflecting the sympathovagal balance, indicating the instantaneous synchronicity of the sympathetic and vagal nerve activities [24,28,29]).

### 2.4. Self-Report Questionnaires

#### 2.4.1. The Sensory Responsiveness Questionnaire-Intensity Scale

The Sensory Responsiveness Questionnaire-Intensity scale (SRQ-IS) [26] was used for group placement. Comprising 58 items, the SRQ is aimed at identifying sensory modulation difficulties through patterns of behavioral responses to daily sensations. Each item involves one sensory stimulus in a single modality including vestibular, auditory, olfactory, visual, gustatory, and somatosensory stimuli, excluding pain. Participants are required to rate the intensity of the aversive/hedonic responses, using a response scale ranging from not at all (1) to very much (5). Content, criterion and construct validity, as well as internal consistency (Cronbach α = 0.90–0.93) and test-retest reliability (*r* = 0.71–0.84; *p* < 0.001–0.005) have been reported [26]. Two scores are derived for each of the two sensory modulation difficulties subtypes: SOR sub-type is identified by applying the SRQ-Aversive sub-scale score (32 items), for scores which are higher than the normal mean cut-off score +2 SD (i.e., 1.87 + 0.52). In this study, this score was used for group placement. The sensory under-responsivity subtype is determined by applying the SRQ-Hedonic sub-scale score (26 items), for scores which are higher than the normal mean cut-off score +2 SD (i.e., 2.10 + 0.66).

#### 2.4.2. The Spielberger State-Trait Anxiety Inventory Questionnaire

The Spielberger state-trait anxiety inventory questionnaire (STAI) [30] assesses anxiety in general (trait) and at the current moment (state). Thus, this questionnaire comprises two sub-scales, consisting of 20 statements each. Subjects are required to rate each statement on a 4-point Likert scale ranging from not at all (1) to very much (4). In this study we utilized only the trait sub-scale.

#### 2.4.3. The Short Version of the Brief Symptom Inventory

The short version of the Brief Symptom Inventory (BSI) [31] is a 13 item self-report screening instrument of psychological distress representing one factor in the Symptom Check List (SCL-90). The BSI quantifies somatization, namely the frequency of complaints or symptoms in different body sites often included in pain evaluation (e.g., chest pain, low back pain, headache, numbness or flushes, vomiting, dizziness).

### 2.5. Procedure

**Figure 1:**
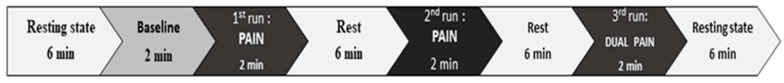
The study design.

The participants completed all the questionnaires and underwent familiarization with the pain stimulus and pain rating scale (i.e., NPS). Thereafter, the pain-50 temperature was determined for each participant and three ECG electrodes were placed and connected to the SUEmpathy 100 device. The tests were conducted as follows: a 2 min baseline activity phase followed by 2 runs of the pain stimuli (i.e., reactive phase) given at pain-50 intensity (1st and 2nd run), and a 3rd run comprised of the dual stimulation. Each run lasted 2 min with an inter-run-interval of 6 min. Recovery was recorded over 6 min. under no stimulus condition (i.e., resting state) and compared to a 6 min resting state recorded initially. The study design is presented in Figure 1.

### 2.6. Data Analysis

Statistical analyses were performed with SAS^®^ V9.4 (SAS Institute, Cary, NC, USA). Data were summarized with descriptive statistics by data type. Groups were compared using a two-sample t-test for continuous variables or ANOVA adjusting for age, after confirming that all variables were normally distributed using the Kolmogorov–Smirnov test. Pearson partial correlation coefficients (age adjusted) were calculated between the variables in each of the groups. Correlation coefficients were compared between the groups (after Fisher’s z transformation) with a Z-test. A 2-sided 5% level of significance was used. Nominal *p*-values are presented. Since this is an exploratory study, no corrections for multiple testing were applied.

## 3. Results

Statistically significant group differences (mean (SD)) were found in age (SOR vs. control group 35 (8.13) vs. 26 (4.76) years; *p* = 0.042). No differences were found in the SRQ-Hedonic score (1.93 (0.47); 2.33 (0.33)), years of education, and family status.

### 3.1. Pain Psychophysics

No difference was found between the study and control groups in the individually tailored temperature, i.e., pain-50 temperature (SOR: 50.5 (2.63) °C vs. control: 49.5 (1.17) °C; *p* = 0.146). In addition, there were no statistically significant group differences (SOR vs. control; *p* > 0.05) in the pain ratings for each of the runs: first run (57.9 (24.7); 59.9 (25.9) NPS), second run (59.3 (21.6); 57.6 (22.6) NPS), third run (52.2 (19.1); 50.2 (29.3) NPS).

### 3.2. HRV Assessments

**Figure 2:**
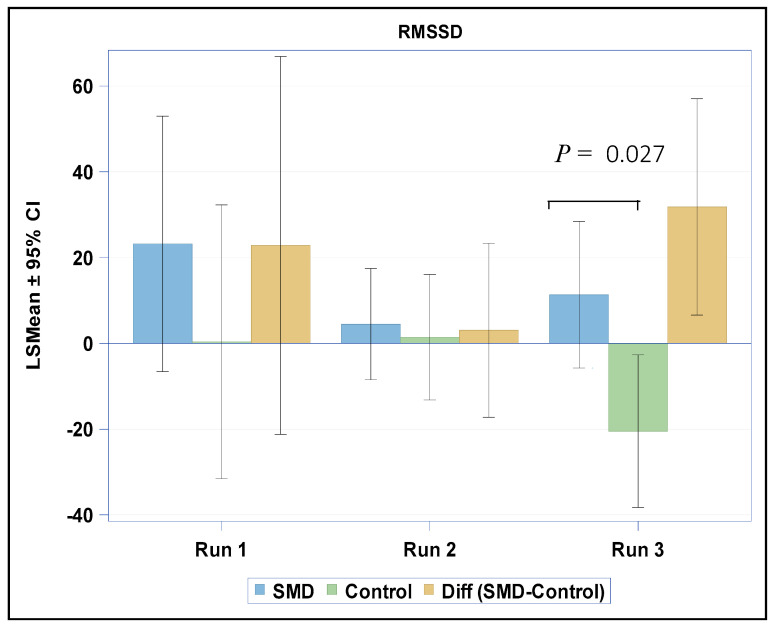
Changes in RMSSD from baseline to reactive phases in both groups. RMSSD: root mean square of successive differences.

When testing for changes in HRV variables between the baseline activity and the reactivity phases, we found a group difference only between the baseline activity phase and the third run of the reactive phase in RMSSD (*p* = 0.027). Within the study group, although the RMSSD was enhanced, there was no statistically significant change (baseline: 52.9 (33.9); reactivity: 60.5 (31.0), *p* = 0.280). Conversely, in the control group, the RMSSD was significantly reduced (baseline: 66.4 (30.3); reactivity: 45.3 (34.2), *p* = 0.029) (Figure 2). No group differences were found in the change between the initial resting state phase and the recovery phase in any of the other HRV variables tested.

### 3.3. Self-Report Measures

Compared to the control group, the study group scored significantly higher on the STAI-Trait score (SOR: 40.22 (9.13) vs. control: 30.88 (1.87); *p* = 0.018). The study group also scored higher (statistical trend only) on the BSI (SOR: 11.12 (7.86); control: 4.44 (3.24); *p* = 0.051).

### 3.4. Partial Correlations (Age Adjusted) between HRV Variables and Self-Report Measures

**Figure 3:**
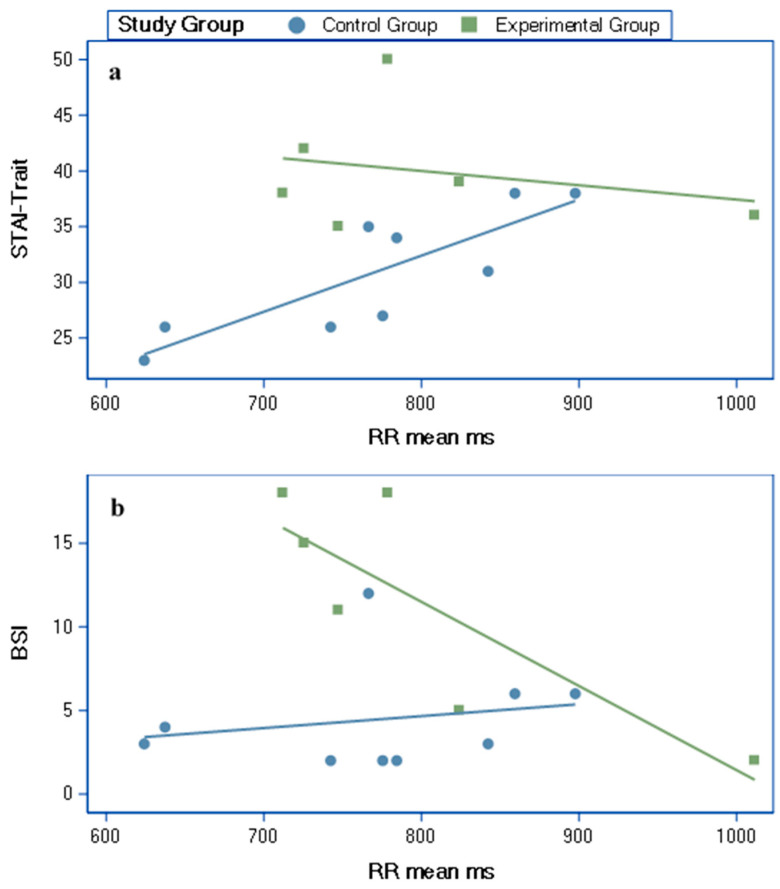
Partial correlations (age adjusted) between RR mean ms and (**a**) the STAI-Trait and (**b**) the short version of the BSI self-report scores. RR Mean ms: R-R interval mean in milliseconds; STAI-Trait: Trait sub-scale of the Spielberger state-trait anxiety inventory questionnaire; BSI: Brief Symptom Inventory.

The R-R mean correlated with both the STAI-Trait and the BSI scores (Figure 3). These correlations showed significant group differences (Table 1, Figure 3).

## 4. Discussion

Children with sensory modulation difficulties were reported as having reduced vagal tone at baseline [11], indicative of poor emotional regulation and reduced behavioral flexibility [32,33]. However, reduced vagal tone at baseline was not identified in our cohort of women with SOR. The normalized vagal tone we found in adults may possibly signify adjusted strategies acquired to cope with environment demands, since research supports the association between vagal tone and coping strategies in order to keep homeostasis [34]. Furthermore, children with sensory modulation difficulties were reported to react with an increase in vagal tone from baseline, instead of withdrawal, in response to a single sensory non-painful stimuli [11]. However, our findings indicate that women with SOR do not demonstrate this response to a single pain stimulus, as reflected behaviorally in similar pain ratings compared to the control group. Thus, women with SOR do not show alterations in their physiological and behavioral self-regulatory capacity, and thus adjust rapidly to a single painful stimulus. However, dual noxious stimulation challenges women with SOR, since no vagal tone withdrawal is evident (i.e., no decrease in RMSSD), unlike the control group, though we found no group difference in pain ratings. Yet, exploring inter subject variability shows a linear association between pain intensity and sympathovagal balance only in the SOR group.

The Neurovisceral Integration Model argues that adaptations to environmental challenges are impacted by environmental, affective, behavioral, cognitive, social and physiological attributes [35,36]. Together, these enable the continued evaluation of threat and safety signs in the environment, as well as any misalignment between the environment and the body’s internal homeostatic processes, in order to produce adaptive behavioral and physiological adjustment [35]. Our results demonstrate that the adaptive behavioral response (pain ratings) did not differ between groups. However, at the physiological level (ANS response), different adjustment responses were found between groups; while controls demonstrated vagal tone withdrawal, in women with SOR, vagal tone withdrawal was not demonstrated. Anchored in the Neurovisceral Integration Model, we assume that the different physiological adjustment in women with SOR enables the adaptive responses to pain stimuli.

Given that pain is a threatening stimulus interrupting the internal homeostasis, mechanisms deriving adaptive responses are required. One of these is the pain inhibits pain mechanism, activated when two painful stimuli are delivered at remote body locations, as was the case in our study. This powerful endogenous analgesic mechanism enables pain inhibition of one stimulus by the other, by activating descending pathways. The vagal nerve can potentially be involved in the activation of the descending pain inhibitory pathways during pain stimulus via the baroreflex. In detail, a painful stimulus activates the spinal nociceptive afferents via the peripheral nociceptors. Simultaneously, pain increases the blood pressure due to increased sympathetic arousal. The rise in blood pressure activates the baroreceptors that are innervated by the vagal afferents which merge in the nucleus tractus solitaries [19]. The nucleus tractus solitaries efferents modulate cardiorespiratory function (i.e., the baroreflex) and also interact with the descending inhibitory pathways [20] in order to coordinate behavioral and physiological responses to pain. Finding no group differences in pain ratings suggests that women with SOR may efficiently activate the pain inhibits pain mechanism as we previously reported [18]. Furthermore, a previous report suggested that in healthy subjects the magnitude of increased sympathetic arousal (i.e., blood pressure) is associated with greater pain inhibition [37]. Therefore, the lack of group differences in the sympathetic variables extracted out of the HRV analysis supports our assumption that in women with SOR, the baroreflex successfully inhibits pain.

We found opposite associations between the level of psychological distress and vagal reactivity (i.e., R-R interval mean) to dual pain stimulation in the SOR group compared to controls. Specifically, while in the SOR group, higher levels of somatization were related to a less efficient vagal inhibitory control over the heart as reflected by short R-R intervals, the control group showed an opposite mild link. The same trend was found for the associations between the anxiety trait and R-R intervals, demonstrating a moderate negative link in the SOR group and an opposite strong significant link in the control group. This suggests that testing inter subject variability demonstrates that high levels of psychological distress in individuals with SOR are associated with less vagal tone reactivity in response to sensory load (i.e., dual pain stimulation), indicative of a disruption of homeostasis and greater stress reactivity [33]. Indeed, high level of anxiety, as was found in our sample of women with SOR and in previous reports [38,39,40], is associated with increased sympathetic cardiac responsiveness [41].

This is an exploratory study including women only, with a small sample size which is the major limitation and warrants further validation with a greater sample size. However, the results of this study may help with better powering future studies that will replicate and validate the results. These studies should also include male subjects in order to generalize the findings.

To summarize, this exploratory study suggests that during dual painful stimulation, women with SOR failed to withdraw vagal tone. Furthermore, examining the inter subject variability within the SOR group, we found that those with a higher psychological distress showed less vagal tone reactivity; also within this group, the sympato-vagal balance was found to be associated with higher pain ratings. It is therefore theorized that in SOR maladaptive response to environmental sensory demands, as expressed by an altered ANS activity, may prevent efficient modulation of painful stimuli over time, resulting in an amplified pain experience, as previously reported in SOR [18]; however, this warrants further research.

## Figures and Tables

**Table 1:** Partial correlation coefficients (adjusted for age) between the R-R mean ms and the psychological distress measures, within and between groups.

	HRV Variables	Experimental Group	Control Group	Between Group Comparison
		r	r	*p*
STAI-Trait	RR mean ms	−0.59	0.84 *	0.006
BSI	RR mean ms	−0.98 *	0.22	<0.001

HRV: heart rate variability; STAI: state-trait anxiety inventory; BSI: Brief Symptom Inventory; * *p* < 0.01.
